# Cellular Restriction Factors of Feline Immunodeficiency Virus

**DOI:** 10.3390/v3101986

**Published:** 2011-10-21

**Authors:** Jörg Zielonka, Carsten Münk

**Affiliations:** 1 Clinic for Gastroenterology, Hepatology and Infectiology, Medical Faculty, Heinrich Heine University, Düsseldorf 40225, Germany; E-Mail: Joerg.zielonka@roche.com; 2 Roche Glycart AG, Schlieren 8952, Switzerland

**Keywords:** FIV, antiviral, restriction factor, APOBEC3, TRIM5, tetherin

## Abstract

Lentiviruses are known for their narrow cell- and species-tropisms, which are determined by cellular proteins whose absence or presence either support viral replication (dependency factors, cofactors) or inhibit viral replication (restriction factors). Similar to *Human immunodeficiency virus type 1* (HIV-1), the cat lentivirus *Feline immunodeficiency virus* (FIV) is sensitive to recently discovered cellular restriction factors from non-host species that are able to stop viruses from replicating. Of particular importance are the cellular proteins APOBEC3, TRIM5α and tetherin/BST-2. In general, lentiviruses counteract or escape their species’ own variant of the restriction factor, but are targeted by the orthologous proteins of distantly related species. Most of the knowledge regarding lentiviral restriction factors has been obtained in the HIV-1 system; however, much less is known about their effects on other lentiviruses. We describe here the molecular mechanisms that explain how FIV maintains its replication in feline cells, but is largely prevented from cross-species infections by cellular restriction factors.

## 1. Introduction

In many species of Felidae, including the domestic cat, individuals may be infected with a unique strain of feline immunodeficiency virus (FIV), and a related virus is also found in African hyenas (for a review, see [1,2]). Viruses isolated from various felid species show monophyletic proviral sequences, which supports the idea that FIVs are not frequently transmitted between different felid species [2–4]. FIV was introduced into the existing felid lineages at several different times during evolution. A co-adaptation between FIV and Felidae is assumed to have occurred for at least 10,000 years, and possibly as much as a million years for some species [2,3]. In contrast, FIV infection of the domestic cat appears to be of more recent origin [2]. In addition, singular events of FIV felid-to-felid cross-species transmissions and repeated FIV transmissions from bobcats to pumas have been observed (for an overview, see [1,5,6]).

In mammals, including felids, the innate immune system provides a first line of defense against pathogens. Induction of type I interferon regulates the expression of several interferon-stimulated genes (ISGs) whose protein products have direct antiviral properties. A group of proteins with potent antiviral properties, known collectively as “restriction factors,” are constitutively expressed in cells, but also induced by type I interferon. These proteins are able to limit replication by targeting specific steps in the viral life cycle [7]. However, lentiviruses have developed counteracting proteins to survive and replicate in their host mammal. While orthologous cellular proteins, which differ specifically in the interaction sites of the counteracting viral proteins, limit inter-species transfer, a high genetic variability in the restriction factors might also limit the spread and extent of infections and disease within the natural host population (intra-species transfer) [8].

FIV infection of felids shows substantial variation in disease outcome: in domestic cats, FIV can induce a strong immune pathology with a T cell depletion and immunodeficiency, resulting in opportunistic infections as well as weight loss and neurological disease (for review see [9,10]); however, in non-domestic felids infections with FIV appear to be apathogenic in most individuals [1]. For example, in FIV-infected lions and pumas, T cell depletion without disease has been described [11,12]. Interestingly, different subtypes of lion FIV may have different patterns of pathogenicity and transmissibility among African lions [13]. Some of these variations are likely influenced by differences in viral adaptation to the restriction factors of their hosts.

Much of today’s knowledge of anti-lentiviral restriction factors is derived from the intensive characterization of the interaction of human immunodeficiency virus type 1 (HIV-1) with different host cells. However, recent investigations have also focused on how FIV escapes its host species’ own restriction factors. Many of the accessory gene products of HIV-1 counteract cellular restriction factors. For example, the Vif protein inhibits APOBEC3 cytidine deaminases (see Section 2), while the Vpu protein protects HIV-1 from tetherin (see Section 4). Vpr inhibits a cellular factor that is still unidentified but, interestingly, Vpx, which is closely related to Vpr (found, for example in *Human immunodeficiency virus type 2*, HIV-2) is a potent inhibitor of SAMHD1 (see Section 5). In contrast to HIV-1, FIV has only a *vif* gene and genes such as *vpr*, *vpx* and *vpu* are not present. Instead, a gene for the less well-characterized multifunctional protein OrfA (Orf-A, Orf-2) is uniquely found in FIV (see Section 6). A further restriction factor, TRIM5α, is not counteracted by accessory proteins. While all primate lentiviruses evolved in the presence of TRIM5α, the feline lentiviruses did not face this protein in their natural hosts because felids express a truncated, inactive variant of the TRIM5 protein (see Section 3) [14].

The strain-specific evolution of FIVs is likely driven by genetic differences in cellular dependency and restriction factors, in addition to selection by the immune system. We can assume that, besides differences in these factors, the FIV cross-species transfer from a felid to a felid/non-felid is also impaired by other limitations. Since FIV infection in domestic cats induces a fatal immunodeficiency that is very similar to AIDS in humans [9,10], FIV infection of cats not only provides a unique model to investigate the evolutionary role of restriction factors on cross-species transmission, but also virus evolution and its impact on AIDS induction.

## 2. FIV and APOBEC3

**Figure 1.**
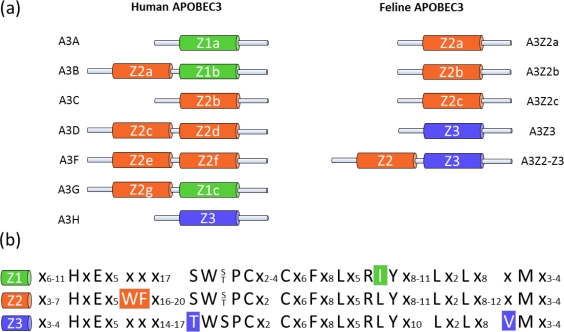
Human and feline APOBEC3 (A3) proteins. (**a**) Schematic representation of human and feline A3 cytidine deaminases. All A3 proteins share at least one zinc (*Z*)-coordinating catalytic motif. The color code indicates the amino acid specificity of the different deaminase domains (Z1, Z2 and Z3). Humans express seven A3 proteins, A3A–A3H; cats express four A3 proteins, A3Z2a–A3Z2-Z3. (**b**) Amino acid sequences of indicated domains. Group-specific distinctions of Z-domains are highlighted.

The mammalian APOBEC3 (A3; apolipoprotein B mRNA editing enzyme catalytic polypeptide 3) protein family was discovered through the study of the HIV-1 Vif. HIV-1 virions that are produced in permissive cells are able to infect permissive and non-permissive cells, whereas Δ*vif* virons produced in non-permissive cells are unable to infect target cells [15,16]. These findings suggested the existence of a Vif-sensitive antiviral factor that was later identified as human APOBEC3G (Figure 1a) [17–19]. This interferon-inducible cellular factor is part of the AID/APOBEC gene family, which share a characteristic zinc (Z)-coordinating catalytic motif (His-X-Glu-X_23–28_-Pro-Cys-X_2–4_-Cys) [20]. The A3 proteins can be classified according to the presence of a Z1, Z2 or Z3 motif (Figure 1b) [21–23]. A multiplicity of evolutionary events driven by host adaptation to viruses—including preservations, deletions, duplications, subfunctionalizations and neofunctionalizations—have led to a number of different forms of A3 genes within mammals [21,23]. Humans express seven A3 proteins (APOBEC3A, -B, -C, -D, -F, -G and -H, Figure 1a) with either one or two zinc-coordinating domains that can inhibit various retroviruses, endogenous retroelements and DNA viruses [24–28].

In the domestic cat, there are three copies of a Z2 gene and a single copy of a Z3 gene (Figure 1a) and are likely present in other feline species as well [23,29,30]. Besides these single-Z-domain proteins, Felidae express three very similar two-domain A3 proteins (Figure 1a, shown as a single A3Z2-Z3 protein for simplicity) by a complex process of read-through transcription and alternative splicing [23,29,30]. A feline A3 gene encoding a Z1 domain protein does not exist. In cats, the A3 genes are polymorphic and are under positive selection, indicating that they are relevant in the “arms race” between host and retrovirus [23].

**Figure 2.**
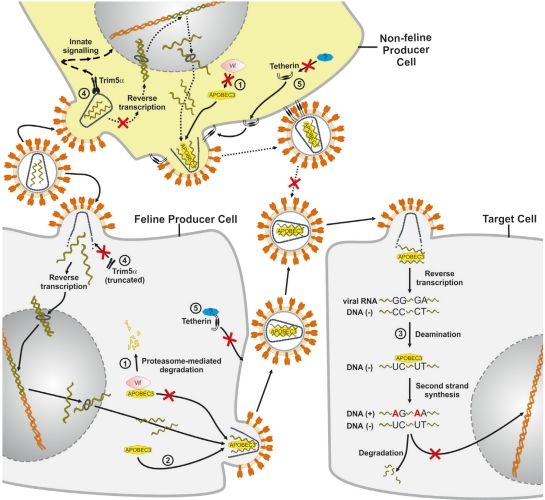
Impact of the cellular restriction factors APOBEC3 (A3), TRIM5α and tetherin on FIV replication. The FIV replication cycle starts by infection of either feline or non-feline cells and the resulting FIV particles then infect a target cell that shows no TRIM5α restriction. FIV Vif targets the feline A3 protein for proteasomal degradation but it is inactive against A3 proteins from non-felid species ①. If Vif is not expressed or does not bind to A3, A3 is packaged into FIV virions budding from the producer cells ②. During the next round of infection in target cells, encapsidated A3 proteins act as inhibitors of virus replication. Single-stranded viral DNA serves as a substrate for A3-induced cytidine deamination, causing hypermutations of the viral DNA, during which deoxycytidines are converted to deoxyuridines. Uracil-containing minus-strand DNA can be targeted by uracil DNA glycosylase, which could lead to endonucleolytic cleavage ③. The number of integrated, highly mutated proviruses is low. In non-feline cells, TRIM5α restricts FIV soon after post-entry, likely by acceleration of the viral uncoating process. TRIM5α may also promote innate immune signaling. The truncated feline TRIM5α is incapable of restricting retroviruses ④. Feline tetherin is not able to restrict direct cell-to-cell spread of FIV, but can restrict the release of cell-free particles. A FIV antagonist (indicated by a question mark) of feline tetherin has not yet been detected. FIV is also inhibited by non-felid tetherin proteins ⑤. Many details of this model are based on experimental results of HIV-1 in primate cells.

The enzymatic activity of virion-encapsidated A3 results in the hydrolytic deamination of cytosine bases in the single-stranded viral (−) DNA that is synthesized during reverse transcription, leading to viral genome degradation, or to G-to-A hypermutations on the (+) DNA strand (Figure 2) [31–36]. However, A3 proteins also have deaminase-independent antiviral activities, and deaminase-deficient A3 mutants are still able to reduce accumulation of reverse transcription products [37–41]. The Vif protein of HIV-1 forms an interaction between A3 and a ubiquitin E3 ligase complex consisting of elongin B and C, cullin5, and ring box-1 [42]; this interaction results in the A3 polyubiquitination that leads to proteasome-mediated degradation of the A3 protein [42–46]. In addition, Vif of HIV-1 inhibits A3 proteins by other non-degrading mechanisms (for a review, see [24]). Like HIV-1, FIV also encodes a Vif protein [23,29,30,47]. FIVs with an inactivated *vif* gene do not productively infect feline blood cells or show replication in cats [48–50].

Recent studies show that FIV Vif counteracts the feline A3 antiviral activity [23,29,30,47]. *vif*-deficient FIV is moderately inhibited by feline A3Z3 and is strongly suppressed by feline A3Z2-Z3, whereas wild-type virus infectivity is not influenced by feline A3 proteins [23,30]. Both FIV and FIVΔ*vif* are very sensitive to three of the seven human A3s (A3F, A3G and A3H) [23,30,47]. The antiviral activity of feline A3s against FIVΔ*vif* correlated with the detection of cytidine deaminated, edited viral genomes [23]. Whether feline A3s also have non-editing antiviral activities has not yet been investigated. Further, it is unknown why feline A3Z2 proteins do not inhibit FIVΔ*vif* or FIV. However, the feline A3Z2 proteins are active cytidine deaminases and they restrict Bet-deficient *Feline foamy virus*. This feline retrovirus uses its accessory protein, Bet, to counteract the feline A3Z2 proteins via a degradation-independent pathway [23,51]. In contrast, expression of FIV Vif induces the degradation of feline A3s and thus prevents the encapsidation of A3s into FIV particles [29,30,47]. The degradation of feline A3 proteins by FIV Vif occurs efficiently in human cells indicating that this Vif activity does not depend on species-specific host factors [29,30,47]. It has not yet been shown but it is likely that FIV Vif recruits elongin B/C, cullin5, and ring box-1 in human cells as in feline cells to induce polyubiquitination of feline A3s.

Vif proteins can be counteractive, non-active or semi-active against different A3 proteins. Surprisingly, the domestic cat FIV Vif protein can efficiently inhibit A3 restriction factors of three tested Felidae (puma, lion, lynx) and shows a reduced activity to A3s from the tiger [30]. These results indicate that most of the diverse felid A3s of big cats are probably not major determinants that prevent cross-species transmission of FIV from the domestic cat to these closely related animal species. In contrast, FIV Vif is completely inactive against A3 proteins from primates and other non-felid species [23,30,47]. A characterization of the molecular interaction of domestic cat FIV Vif with A3s of different felids may reveal the binding domains of Vif and A3; it may also explain the broad activity of domestic cat FIV Vif.

## 3. FIV and TRIM5

**Figure 3.**
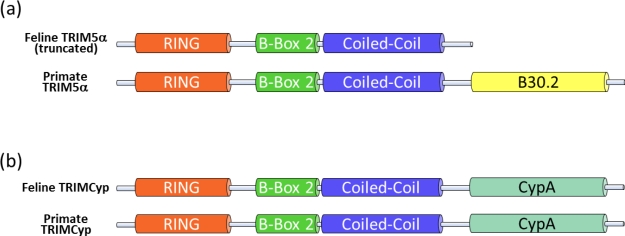
Domain structure of TRIM5α and TRIMCyp proteins. (**a**) TRIM5α is defined by four domains: RING finger, B-box 2, coiled-coil and B30.2. The feline TRIM5α lacks the B30.2 capsid-binding domain because of a premature stop codon in the mRNA transcript. (**b**) In some monkeys, TRIMCyp is expressed, in which the B30.2 domain of TRIM5α is replaced with a cyclophilin A (CypA) domain. Expression of feline TRIMCyp was not observed. Dietrich *et al.* produced a synthetic feline TRIMCyp by fusion of the truncated TRIM5α and feline cyclophilin A [75].

HIV replication in simian cells is blocked during uncoating at the early post-entry stage—a finding that was used to identify the restriction factor TRIM5α [52], which is constitutively expressed, but interferon treatment can further increase its levels [53]. TRIM5α is a tripartite motif protein with a RING, B-box 2 and coiled-coil (CC) domain (RBCC), as well as a carboxy-terminal B30.2 (SPRY) domain (Figure 3a). The B30.2 domain of TRIM5α binds to the viral capsid of incoming viral particles, while the RBCC domains mediate the localization to cytoplasmic bodies and are important for TRIM5α self-association [54–61]. TRIM5α inhibits HIV-1 and other retroviruses in a species-specific manner. Thus retroviruses replicate in the presence of TRIM5α proteins of their host species and are inhibited by orthologous TRIM5α proteins. Species-adapted retroviruses evolved viral capsids that do not bind TRIM5α proteins expressed in their host species [62]. Simian TRIM5α likely acts at several levels: it restricts HIV-1 by accelerating the viral uncoating process, which leads to an inhibition of the reverse transcription (Figure 2) [63–65], and TRIM5α also leads to an integration block that appears after the reverse transcription block is relieved by inhibition of the proteasome [66]. Insights into the multifunctional nature of TRIM5α were obtained in a recent study by Pertel *et al.* that suggests that TRIM5α is a pattern-recognition receptor for HIV-1 (Figure 2) [67]. TRIM5α promotes innate immune signaling of the mitogen-activated protein kinase and NF-κB signaling pathways by its association with the retroviral capsid lattice and the E2 ubiquitin-conjugating enzyme complex UBC13-UEV1A that activates TAK1 kinase [67]. Furthermore, the authors demonstrated that TRIM5α has a specific effect on the expression of NF-κB- and AP-1-responsive inflammatory chemokines and cytokines [67]. The knockdown of TRIM5α in myeloid cells attenuates lipopolysaccharide (LPS)-induced immune signaling and also rescues viral infections from the LPS-induced antiviral gene expression [67]. Thus, TRIM5α plays a role in LPS-triggered immune activation through the Toll-like receptor 4 pathway.

Human and rhesus macaque TRIM5α can inhibit the infection of FIV if expressed in feline cells [54,68]. In single-round infection assays, it appears that the antiviral activity of the rhesus protein is stronger than that of human TRIM5α. Feline cells expressing human or rhesus TRIM5α also potently block spreading replication of FIV without the appearance of revertants [68]. Most human cell lines, with the exception of HEK 293T, are not permissive for transduction by FIV vectors, especially T cell lines that show a strong restriction [68].

Owl monkeys and macaques express a protein called TRIMCyp due to a cDNA of the cyclophilin A (CypA) gene that retrotransposed into the 3′ region of the TRIM5 gene [69–73]. In owl monkeys, the CypA cDNA is inserted into intron seven of the TRIM5 gene [71]. A distinct evolutionary origin of TRIMCyp is found in three macaque species (*M. fascicularis*, *M. mulatta*, and *M. nemestrina*) where the CypA cDNA retrotransposed in the 3′ untranslated region of the TRIM5 gene and macaque TRIMCyp is expressed by exon skipping from exon six to CypA [69,70,72,73]. Owl monkey as well macaque TRIMCyps consist of the RBCC domains of TRIM5 fused with a carboxy-terminal CypA moiety (Figure 3b) [69–74]. CypA binds the surface of the capsid proteins of HIV-1 and FIV that form the viral core [75–78]. In FIV, the interaction of CypA can be prevented by a P90A mutation in the loop between helix 4 and 5 of the capsid [75,78]. TRIMCyp of Owl monkeys and macaques, or an artificial human TRIMCyp, restricts infection with FIV [54,72,73,78–82]. Similar to TRIM5α, treating rhesus TRIMCyp-expressing feline cells with a proteasome inhibitor relieves the block in the reverse transcription of FIV, but does not restore the capacity of FIV to transduce these cells [73].

McEwan *et al.* discovered that *Feliformia* express a truncated TRIM5 gene, which explains why feline cells do not show a TRIM5-typical restriction to retroviruses (Figure 2) [14]. The feline mRNA of TRIM5 contains a premature stop codon expressing a RBCC protein without the B30.2 domain (Figure 3a). In cats, the missing B30.2 domain is not replaced by CypA as seen in some monkeys [14]. The truncated feline TRIM5 appears to be without any antiretroviral activity as overexpression of the feline TRIM5 does not prevent infection with murine leukemia virus, HIV-1 or a simian immunodeficiency virus (SIV) of macaques (SIVmac) [14]. However, it is possible that the truncated feline TRIM5 protein is similar to the human TRIM5α involved in LPS-mediated signaling [67], potentially explaining why this gene is retained in Feliformia. Interestingly, a synthetic fusion of the feline TRIM5 to the feline CypA (Figure 3b) generated a potent inhibitor of FIV and HIV-1 [75,83]. These results show that the RBCC domains of feline TRIM5 retain their intrinsic antiviral function.

## 4. FIV and Tetherin

The restriction factor tetherin (also known as CD317 or BST-2 or HM1.24), was identified by analyzing the cell type-specific particle release block of HIV-1 deficient for the viral protein Vpu [84,85]. Tetherin is an interferon-induced protein that “tethers” HIV-1Δ*vpu* particles at the cell surface (Figure 2). The Vpu protein of HIV-1 interferes with the cell surface expression of human tetherin in part by inducing its degradation [86,87]. Primate lentiviruses that lack a *vpu* gene use either their Nef or Env proteins to counteract tetherin in the host cells (for review see [88–90]). In general, the lentiviral proteins that counteract are only active against tetherin proteins of their own host species. In addition to retroviruses, tetherin proteins are also inhibitory to herpesviruses, arenaviruses, filoviruses, rhabdoviruses, orthomyxoviruses and paramyxoviruses [91–98].

**Figure 4.**
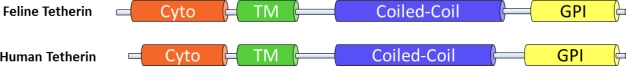
Schematic structure of feline and human tetherin. Tetherins from both species share their principal elements: Cytoplasmatic (Cyto)-, transmembrane (TM)-, coiled-coil- and glycophosphatidyl-inositol (GPI)-anchor domains.

Tetherin is an unusual type II integral membrane protein (Figure 4) with an N-terminal transmembrane domain, an extracellular CC domain and a C-terminal glycosylphospatidylinositol (GPI) lipid anchor at its C-terminus [99]. Recent data indicate that the GPI anchor motif functions as a second transmembrane motif [100]. In humans, tetherin is expressed on several specialized cell types such as hepatocytes, monocytes, epithelial cells, terminally differentiated B cells and bone marrow stromal cells. It is upregulated in many cell types upon treatment with interferon [101]. The feline tetherin protein was recently cloned and functionally characterized [83,102]. Similar to human and mouse tetherin, the expression of feline tetherin is inducible by interferons but a detailed tissue expression pattern has not yet been determined [83,102]. In a transient co-expression system, feline tetherin is a potent inhibitor for the release of FIV and HIV-1 particles [83]. The release of FIV virus-like particles and wild-type particles is also strongly inhibited by the human tetherin [83,91]. Apparently, under such experimental conditions, FIV has no viral determinant that inhibits the antiviral activity of feline tetherin. In contrast to transient expression of tetherin, stably expressing feline tetherin in feline CrFK cells did not restrict virus spread of FIV (human tetherin was not tested) [83]. Similarly, in the HIV-1 system, the cell-to-cell transfer of *vpu-*deficient HIV-1 in T cells was not inhibited by human tetherin [103], although, contrasting results have also been reported [104]. This might indicate that Vpu of HIV-1 is not strongly required for viral spread *in vivo*. Support of this model comes from the observation, that in contrast to Vpu of HIV-1 pandemic group M, the Vpus of non-pandemic AIDS inducing HIV-1 groups O and N show no, or very low activity, against human tetherin [105]. These findings potentially explain why HIV-1 group O and N viruses propagate less efficiently in the human population than the pandemic HIV-1 group M virus. Together, these data indicate that FIV, in contrast to HIV-1, is transmitted mainly as a cell-associated virus and not as a cell-free virus.

## 5. FIV and SAMHD1

Human cells of the myeloid lineage such as blood monocytes or monocyte-derived dendritic cells (mDCs), are highly refractory to infection by HIV-1 due to a post-entry restriction [106–111]. Some primate lentiviruses, such as SIV endemic to sooty mangabeys (SIVsm), SIVmac and HIV-2, do not show this replication restriction (for a review, see [112]). Human and feline mDCs also show a resistance against the productive infection with FIV [113–115]. In contrast to HIV-1, lentiviruses such as SIVsm/SIVmac/HIV-2 express an accessory protein called Vpx that is specifically encapsidated into viral particles [116,117]. Loss of Vpx expression in SIVmac and HIV-2 has no effect on the viral replication in established cell lines, but Vpx mutants show a delayed replication in cultures of PBMCs (peripheral blood mononuclear cells) and a replication block in macrophages [118–120]. *In vivo*, SIVsm (isolate PBj) needs Vpx expression for efficient dissemination and the acute, unique pathogenesis of SIVsmPBj [121]. In infected monkeys, SIVmac Vpx mutants show a lower viremia, a delayed CD4 cell decline and a later AIDS induction compared to wild type viruses [122]. Thus, a Vpx-function for AIDS induction by SIVmac is not required and it is unclear why only some lentiviruses encode Vpx and infect mDCs.

**Figure 5.**
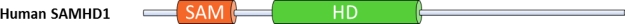
Schematic representation of the human SAMHD1 protein indicating the SAM and the HD domain. A putative feline SAMHD1-encoding gene is found on cat chromosome 13.

In myeloid cells, Vpx mutants of HIV-2 and SIVmac show a post-entry block at reverse transcription or uncoating [115,123–125]. The Vpx protein forms a cullin 4-based E3 ubiquitin ligase complex (for a review, see [112]) indicating that, similar to Vif, Vpx acts as a substrate receptor to induce the degradation of a cellular protein. Recently, the human protein SAMHD1 was identified to be causative for the post-entry restriction of HIV-1 in myeloid cells [126,127]. Viral particle-associated Vpx protein induces a proteasome-dependent degradation of SAMHD1 in the target cell very early after entry [126,127]. The SAMHD1 protein has two domains that are widely found in all genomes: an N-terminal SAM domain followed by a HD domain (Figure 5). SAM (sterile α motif) domains have diverse functions, such as binding to kinases, other proteins or RNA [128]. The HD domain with histidine and aspartic acid residues for metal coordination defines a superfamily of metal-dependent phosphohydrolases, which includes many proteins that are involved in nucleic acid metabolism such as dGTPases, nucleotidyltransferases and helicases [129]. Mutations in the SAMHD1 gene lead to Aicardi-Goutieres syndrome, which includes cerebral atrophy, leukoencephalopathy, hepatosplenomegaly, and increased production of α-interferon [130]. The antiviral mechanism of SAMHD1 is not yet identified but, based on the activities of proteins that share homology with SAMHD1, it is likely that SAMHD1 acts either as a nuclease that destroys the viral genetic material of incoming viruses, or functions as a sensor (or sensor-associated protein) that induces antiviral proteins. It would be interesting to determine whether the feline gene for SAMHD1 (found on cat chromosome A3) encodes an antiviral protein, and whether FIV expresses a viral counteracting factor against feline SAMHD1 that functions similarly to Vpx.

## 6. Major Challenges in Research on FIV Restriction Factors

The HIV-1 origin in humans is one of best studied lentiviral cross-species transmissions [131]. Because of the very high genome sequence identity between the two Hominidae, *Pan troglodytes* and *Homo sapiens*, it is not surprising that SIV of chimpanzees (SIVcpz) quickly adapted and developed a pandemic distribution once introduced in the human population. However, the evolution of HIV-2, derived from a SIV endemic to sooty mangabeys (SIVsm) demonstrates that lentiviruses can also rapidly cross between species in different families, in that case from Cercopithecidae to Hominidae [132,133]. FIV cross-species transfers are described so far only in Felidae, and mostly as singular events, with the exception of repeated transmissions of FIVs from bobcats to pumas [1,5,6]. It is unknown what the impact of restriction factors *versus* the relevance of other factors are in these cross-species transmissions [2]. There is a need for more field and laboratory studies that quantitate the potential of molecularly defined feline lentiviruses to establish cross-felid infections. It would be interesting to learn whether these cross-species transmitted viruses replicate *in vivo* to similar levels, with similar tissue distributions as the species-adapted FIVs, and whether they are rapidly controlled by the innate and/or adaptive immune system of the cat. Even for regular FIV infections in the domestic cat, information on the importance of cellular restriction factors is limited. Modern methods to knock-down the expression of genes or genetically modify FIV target cells with novel restriction factors (to model human HIV-1 gene therapy) will be useful in gaining further insights into the evolutionary potential of viral adaptation or its intrinsic constraints, the stability of viral genes and genomes, and the impact of restriction factors on the health of infected animals. A recent breakthrough is the generation of rhesus TRIMCyp transgenic cats [82]. PBMCs of theses transgenic animals expressed variable amounts of rhesus TRIMCyp and showed a partial resistance to FIV replication [82].

Besides the impact of known restriction factors on FIV replication and their use in therapeutic models, FIV has not been utilized in a systematic search for antiviral proteins and dependency factors. The recent identification of novel restriction factors that are counteracted by accessory proteins of HIV-1 raises the question as to whether FIV might also be used to identify novel cellular restriction factors. In this regard, it might be interesting to note that FIV encodes the protein OrfA, whose function is still enigmatic [134,135]. OrfA does not counteract of feline tetherin [83], but is important for replication *in vivo* and in primary T cells, and its exact role in FIV biology is not yet established. Different studies describe various functions of OrfA including: transactivating viral transcription, regulating the infectivity of FIV particles, inducing G2 cell cycle arrest, and downregulating the FIV receptor CD134 [136–139].

The induction of interferon following FIV infection of feline PBMCs and cats suggests that pattern-recognition receptors in hematopoietic cells detect the FIV infection [140]. How FIV or other lentiviruses are sensed, whether FIV has evolved partial escape mechanisms of this detection, and which feline ISGs show anti-FIV activity need much more investigation. A systematic analysis of all ISGs with FIV and FIV deleted in specific genes could identify factors that are antiviral and are counteracted by FIV’s proteins. In human cells, many interaction partners of A3G, TRIM5α, tetherin and SAMHD1, and of HIV-1 accessory proteins, have been described or will be published soon (e.g., [42,67,141–143]). Thus, systems biology approaches for FIV and feline cells will be important to learn about the conserved and differing pathways used by HIV-1 and FIV. It may also allow us to better utilize the FIV cat model to discover novel treatment and healing options for HIV-1-infected patients.
